# Cibinetide dampens innate immune cell functions thus ameliorating the course of experimental colitis

**DOI:** 10.1038/s41598-017-13046-3

**Published:** 2017-10-12

**Authors:** Manfred Nairz, David Haschka, Stefanie Dichtl, Thomas Sonnweber, Andrea Schroll, Malte Aßhoff, John E. Mindur, Patrizia L. Moser, Dominik Wolf, Filip K. Swirski, Igor Theurl, Anthony Cerami, Michael Brines, Günter Weiss

**Affiliations:** 1 Department of Internal Medicine II, Infectious Diseases, Immunology, Rheumatology, Pneumology, Medical University Innsbruck, Innsbruck, Austria; 2 Center for Systems Biology, Massachusetts General Hospital and Harvard Medical School, Boston, MA USA; 3 Department of Radiology, Massachusetts General Hospital and Harvard Medical School, Boston, MA USA; 4 Department of Pathology, Medical University of Innsbruck, Innsbruck, Austria; 5 Medical Clinic III for Oncology, Hematology, Immunoncology and Rheumatology, University Hospital Bonn, Bonn, Germany; 6 Araim Pharmaceuticals, Tarrytown, New York, United States of America

## Abstract

Two distinct forms of the erythropoietin receptor (EPOR) mediate the cellular responses to erythropoietin (EPO) in different tissues. EPOR homodimers signal to promote the maturation of erythroid progenitor cells. In other cell types, including immune cells, EPOR and the ß-common receptor (CD131) form heteromers (the innate repair receptor; IRR), and exert tissue protective effects. We used dextran sulphate sodium (DSS) to induce colitis in C57BL/6 N mice. Once colitis was established, mice were treated with solvent, EPO or the selective IRR agonist cibinetide. We found that both cibinetide and EPO ameliorated the clinical course of experimental colitis in mice, resulting in improved weight gain and survival. Correspondingly, DSS-exposed mice treated with cibinetide or EPO displayed preserved tissue integrity due to reduced infiltration of myeloid cells and diminished production of pro-inflammatory disease mediators including cytokines, chemokines and nitric oxide synthase-2. Experiments using LPS-activated primary macrophages revealed that the anti-inflammatory effects of cibinetide were dependent on CD131 and JAK2 functionality and were mediated via inhibition of NF-κB subunit p65 activity. Cibinetide activation of the IRR exerts potent anti-inflammatory effects, especially within the myeloid population, reduces disease activity and mortality in mice. Cibinetide thus holds promise as novel disease-modifying therapeutic of inflammatory bowel disease.

## Introduction

Erythropoietin (EPO) is a type I cytokine that fulfills dual functions. In its classical endocrine role, EPO produced by the kidney acts as the key regulator of erythropoiesis via inhibition of erythroid progenitor cell apoptosis and stimulation of differentiation^1,2^. Additionally, locally-produced EPO mediates extra-erythropoietic activity in non-hematopoietic cells and tissues^3^. Accordingly, basic research has characterized EPO as a tissue-protective and anti-apoptotic cytokine in animal models of ischemia, ischemia-reperfusion injury or mechanical trauma in various tissues including the nervous system, retina, myocardium, lung, kidney, liver or transplanted pancreatic islets^4–12^. Moreover, it has been shown that EPO ameliorates disease activity in experimental arthritis, encephalomyelitis and colitis in mice, suggesting that EPO exerts anti-inflammatory and tissue protective effects in autoimmune diseases^13–16^.

These pleiotropic effects of EPO are transduced by two different receptor isoforms^3^. The erythropoietic response to EPO is initiated via a receptor formed by two identical EPO receptor (EPOR) subunits, which mediate Janus kinase-2 (JAK2)-dependent activation of signal transducer and activator of transcription-5 (STAT5)^17^. STAT5 subsequently translocates to the nucleus to regulate hemoglobin (Hb) synthesis, control cell cycle progression and inhibit the apoptosis of erythroid progenitors^18,19^. In parallel to the STAT5 pathway, EPOR homodimers activate mitogen-activated kinases (MAPKs) and nuclear factor (NF)-κB^20^. In contrast, the anti-apoptotic and anti-inflammatory effects of EPO are transduced via an alternative receptor composed of subunits EPOR and CD131, the beta common receptor shared by granulocyte macrophage (GM)-CSF, interleukin (IL)−3 and IL-5^21^, also termed the innate repair receptor (IRR). Activation of the IRR is paracrine in nature, requiring high local concentrations of EPO, yet facilitates similar signal transduction events including STAT5 and MAPK activation. The effects of IRR activation on NF-kB function are diverse and may be dependent on the experimental model or cell type studied. For example, EPO protected cultured cerebrocortical neurons form excytotoxic and nitrosative stress through activation of JAK2 and NF-κB, resulting in increased expression of anti-apoptotic gene products^4^. In contrast, *in vivo* models of myocardial and hepatic ischemia/reperfusion injury have shown that EPO treatment protected from tissue damage via inhibition of NF-κB activation^10,22^. Similarly, sensitization to chemotherapeutics by EPO *in vitro* involved the inhibition of both JAK2 and NF-κB^23^.

Cibinetide (also termed ARA290 or pyroglutamate helix B surface peptide (pHBSP)), is a synthetic oligo-peptide rationally designed to selectively activate the IRR^24^. Cibinetide interacts with the external hydrophilic domain of the EPOR/CD131 complex and has been shown to exert benefical effects in animal models of myocardial infarction, dilated cardiomyopathy, experimental ischemic stroke, peripheral nerve trauma and wound healing. Furthermore, randomized placebo controlled trials have demonstrated its safety and efficacy in human subjects affected by peripheral neuropathy secondary to sarcoidosis or diabetes mellitus^25–27^. Cibinetide improves symptoms in patients with sarcoidosis-associated small nerve fiber loss and increases corneal small nerve fiber abundance.

Cibinetide lacks erythropoietic effects as it does not activate EPOR homodimers. Correspondingly, unchanged Hb levels have been reported following repeated applications of cibinetide in patients. Thus, cibinetide is not associated with unintended side effects such as thromboembolism secondary to stimulation of erythropoiesis as ascribed for full length recombinant erythropoietin.

The purpose of this study was to evaluate the therapeutic potential of cibinetide in the model of experimental DSS-colitis and to investigate possible mechanisms that mediate its immuno-modulatory effects. We report that cibinetide had multiform anti-inflammatory properties in inflamed tissues. While cibinetide did not affect erythropoiesis, it reduced the influx of myeloid cells into the lamina propria and inhibited the local production and systemic availability of TNF, IL-6, IL12/IL-23, myeloid derived chemokines and nitric oxide (NO) in mice suffering from DSS colitis. Mechanistically, these effects of cibinetide were transduced via CD131 and JAK2 signaling which resulted in impaired activation of the NF-κB subunit p65 thereby blocking transcriptional activation of NF-κB driven inflammatory mediators. Consequently, cibinetide exerted beneficial effects in DSS-colitis by improving disease activity and survival, recommending cibinetide as a promising therapy for human inflammatory bowel disease.

## Results

### Cibinetide ameliorates the clinical course of dextran sulphate sodium-induced colitis

Full length EPO has anti-inflammatory effects in a model of inflammatory bowel disease, namely DSS-colitis, that are mediated via NF-κB de-activation^16^. We thus questioned whether this effect depended on IRR activation by determining whether the specific IRR agonist cibinetide improved DSS-colitis, where pathophysiology and tissue damage are centrally driven by NF-κB regulated pathways. C57BL/6N mice were therefore administered 3% DSS via the oral route for 7 days (days 1 to 7). From day 8 to 14, mice had *ad libitum* access to drinking water and were injected intraperitoneally daily with either EPO, cibinetide or an equal volume of PBS (solvent).

**Figure 1:**
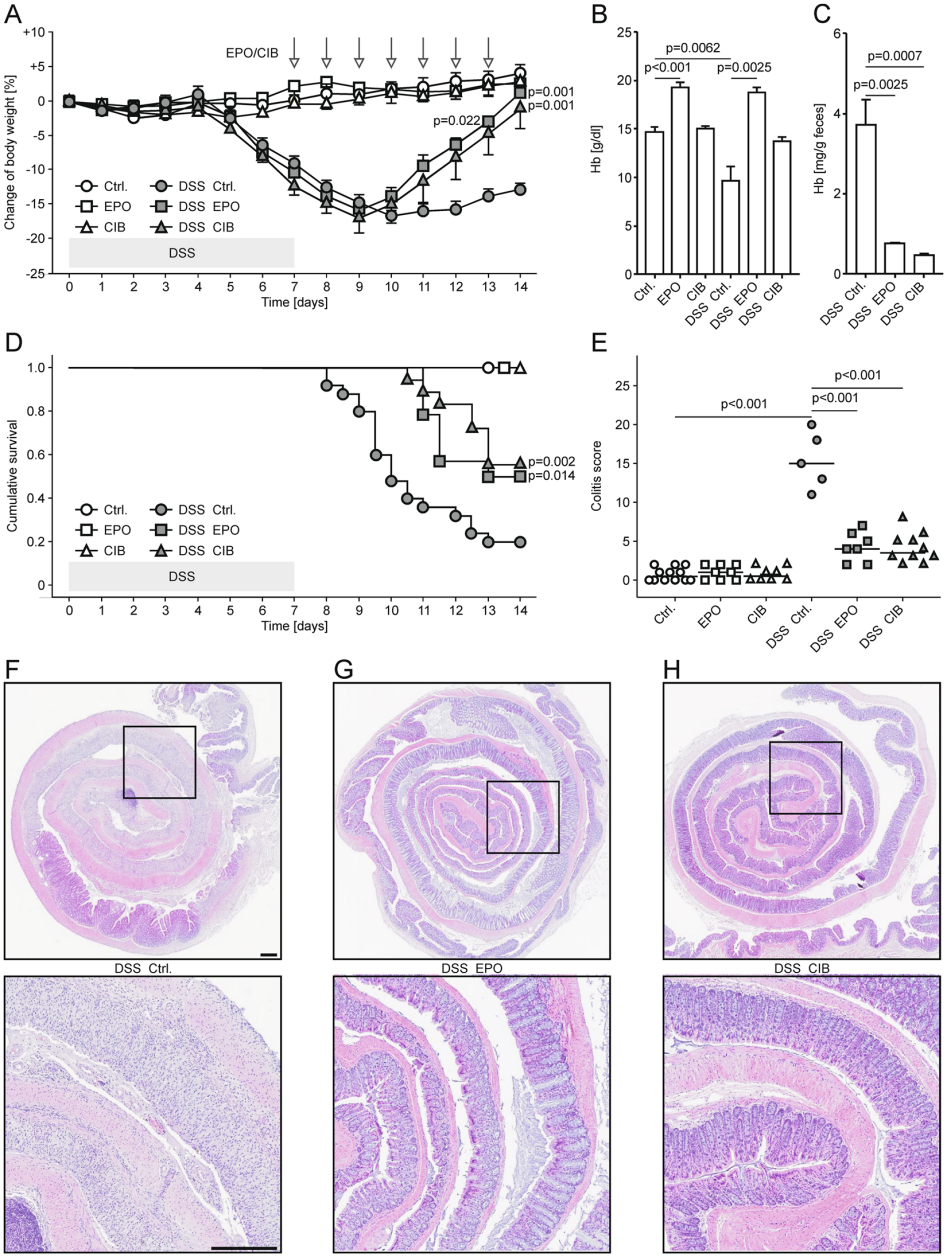
Cibinetide and EPO treatment improve disease activity in DSS-induced colitis. (**A**) C57BL/6N mice received drinking water or 3% DSS from days 1 to 7. Through days 8 to 14, all animals received drinking water. Starting on day 8, mice were daily injected with PBS (Ctrl.; given as solvent), 1.5 pmol/g body weight rhu erythropoietin (EPO) or 1.5 pmol/g body weight cibinetide (CIB) as indicated by arrows. The change in body weight is expressed as percentage of body weight from day 0, and data are shown as means ± S.E.M. for 5–12 mice per group. Results were compared by ANOVA using Bonferroni correction. Statistical significant differences between DSS-mice treated with EPO or CIB in comparison to PBS treatment are indicated next to the corresponding symbol. (**B**) Hemoglobin (Hb) levels of the animals presented in (a) were measured on day 14 by an automated veterinary cell counter. Data were compared by Kruskal-Wallis testing. Data are shown as means ± S.E.M. *n* = 5–12 mice per group. (**C**) Fecal Hb levels were measured on day 14 and normalized for dry weight. Data were compared by Kruskal-Wallis testing. Data are shown as means ± S.E.M. *n* = 5–9 mice per group. (**D**) Survival of DSS-exposed mice treated with PBS, CIB, or EPO was followed for 14 days. Data were compared by log-rank test and are depicted as Kaplan-Meier plot. *n* = 3 per group for mice on drinking water, *n* = 14–24 per group for mice on DSS. (**E**) Histopathological colitis scores of mice receiving drinking water or 3% DSS and subsequently treated with PBS, CIB, or EPO (*n* = 5–12 per group). Each point represents an individual mouse, horizontal bars represent medians. Statistically significant differences between treatment groups as determined by ANOVA using Bonferroni correction are indicated. *n* = 5–12 mice per group. (**F**–**H**) Photomicrographs of HE-stained colonic sections showed mucosal thickening, epithelial hyperplasia and inflammation in PBS-treated DSS-mice (**F**) and nearly unaffected mucosa observed in EPO- (**G**) or CIB-treated DSS-mice (**H**). Colon histology is shown, inserts are indicated. Scale bars: 100 μm.

In comparison to DSS-exposed mice treated with solvent, animals receiving either EPO or cibinetide presented with a significantly improved weight gain (Fig. 1A). Expectedly, EPO-treated animals exhibited increased hemoglobin (Hb) levels (Fig. 1B), whereas cibinetide treatment did not result in such erythropoietic activity *in vivo* or *in vitro*. Specifically, EPO but not cibinetide stimulated the differentiation of murine erythropoietic progenitors (for gating strategy see Supplementary Fig. S1A) in the bone marrow *in vivo* (Supplementary Fig. S1B–S1G) and increased Hb protein and CD71 mRNA levels in human mononuclear cell cultures (Supplementary Fig. S1H and S1I). Cibinetide prevented the occurrence of anemia in DSS-exposed mice (Fig. 1B), possibly by reducing intestinal bleeding as evident from reduced fecal Hb content (Fig. 1C). Importantly, lethality was also significantly lower in the EPO and cibinetide treatment groups, respectively (Fig. 1D). Correspondingly, histopathologic changes as quantified by a colitis score^28^ and immune cell tissue invasion/mucosal thickening were significantly and similarly reduced in colitis mice treated with EPO or cibinetide (Fig. 1E and F–H).

### Cibinetide inhibits immune cell influx into the colonic lamina propria

**Figure 2:**
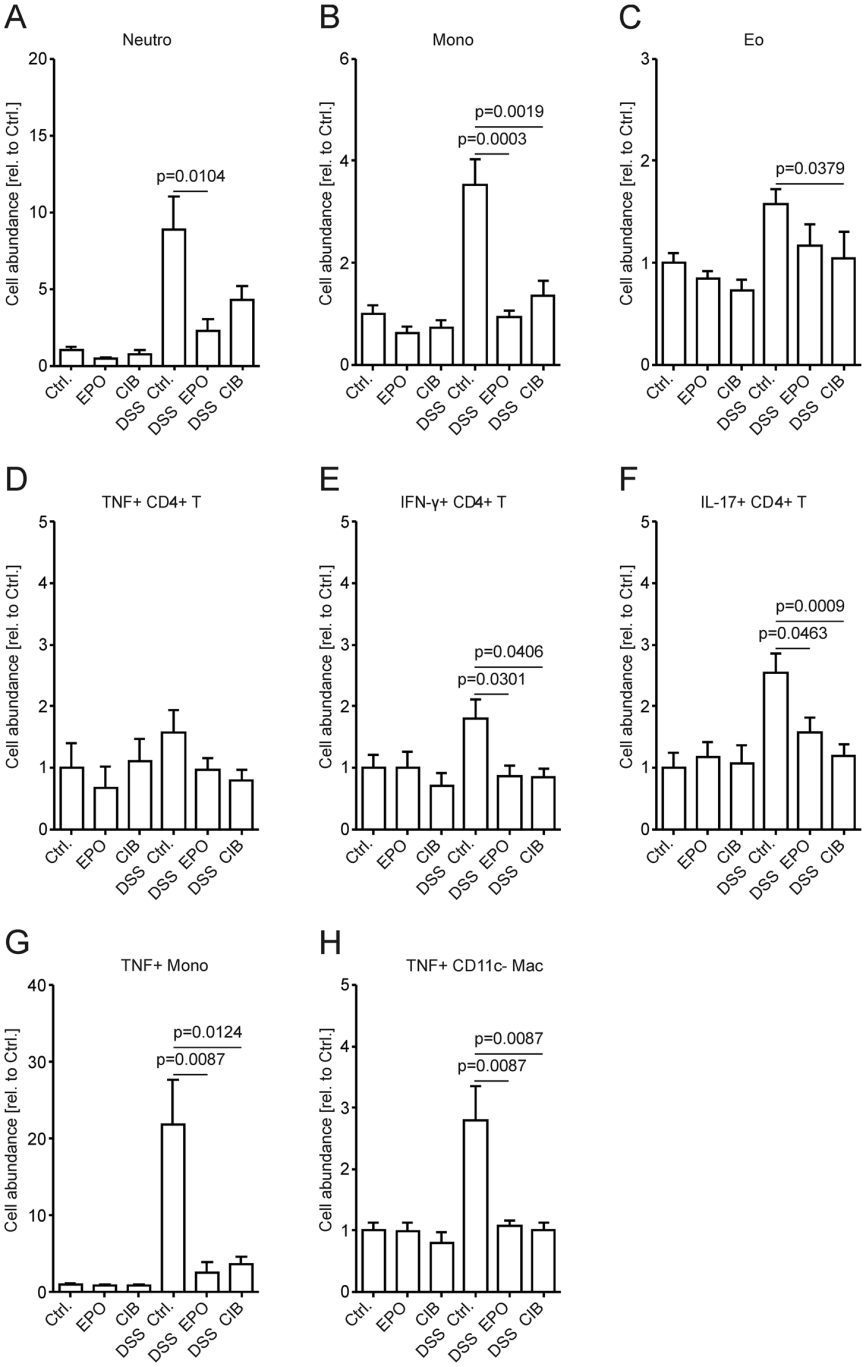
Cibinetide (CIB) or EPO treatment reduces immune cell infiltration into lamina propria. Leukocyte populations in the colonic lamina propria were analyzed on day 14 by flow cytometry. To assess cytokine expression by flow cytometry, single cell suspensions were re-stimulated with PMA and ionomycin in the presence of Golgi blockage. Cells were permeabilized for intracellular staining. Gating strategies are detailed in Supplementary Fig. S2A, S2B and S3A. Neutrophils (**A**), monocytes (**B**), eosinophils (**C**), TNF^+^ CD4^+^ T cells (**D**), IFN-γ^+^ CD4^+^ T cells (**E**), IL-17A^+^ CD4^+^ T cells (**F**), TNF^+^ monocytes (**G**) and TNF^+^ CD11b^+^ CD11c^−^ macrophages (**H**) were quantified. Numbers represent cell abundance relative to PBS-injected controls (Ctrl.) kept on drinking water. Data were compared by Kruskal-Wallis testing. *n* = 5–12 mice per group.

Flow cytometric characterization of immune cells (for gating strategies see Supplementary Fig. S2A, S2B and S3A) infiltrating the lamina propria in response to DSS showed that treatment with EPO or cibinetide resulted in reduced accumulation of neutrophils, monocytes and eosinophils in the colon (Fig. 2A–C). In addition, we noted a significant reduction in CD4^+^ T cell numbers expressing either IFN-γ or IL-17A in the colon of mice having received EPO or cibinetide as compared to control treated littermates (Fig. 2E and F). A substantial reduction of TNF positivity was found in myeloid cells, especially of Ly-6C^+^ monocytes and CD11b^+^ CD11c^−^macrophages (Fig. 2G and H) but not in the CD4^+^ T cell population (Fig. 2D). In addition, either population of myeloid cells displayed reduced intracellular Nos2 staining in the EPO and cibinetide treatment groups (Supplementary Fig. S2C and S2D). No change was observed in intralesional numbers of CD11c^+^ DC (Supplementary Fig. S2E), mast cells (Supplementary Fig. S2F), IL-10^+^ CD4^+^ T, CD8^+^ T or B cells (Supplementary Fig. S3B, S3C and S3D, respectively).

### Lamina propria macrophages express the innate repair receptor and respond to cibinetide

**Figure 3:**
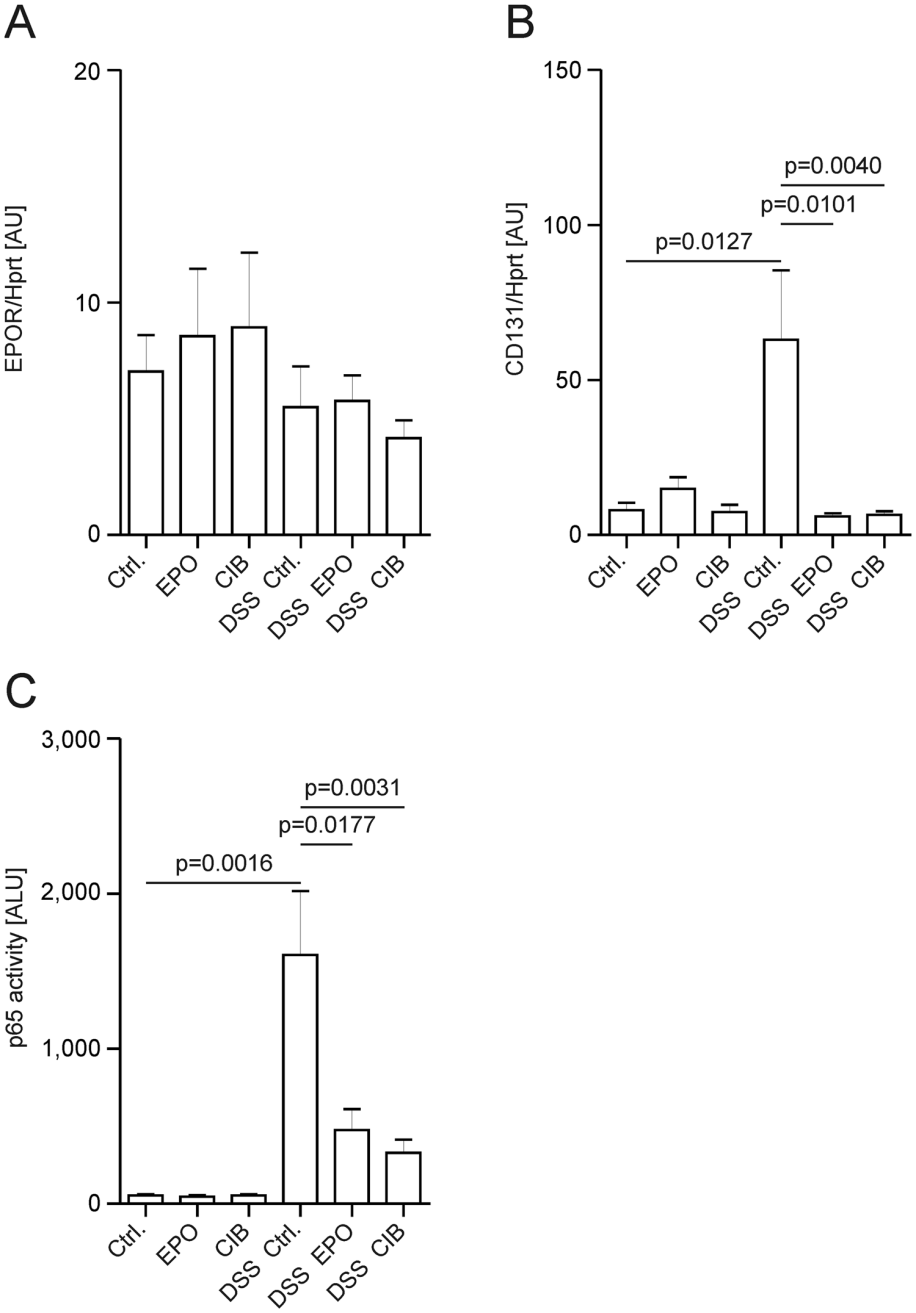
EPO and cibinetide counteract NF-κB p65 activation in lamina propria macrophages. CD11b^+^ CD11c^−^ macrophages were FAC-sorted from the colonic lamina propria on d14 of DSS exposure to prepare total RNA and nuclear proteins. Epor (**A**) and CD131 (**B**) mRNA expression was assessed by qPCR. Activation of p65 (**C**) was measured by an ELISA-based assay. Data were compared by Kruskal-Wallis testing. *n* = 5–12 mice per group.

Next, we FAC-sorted CD11b^+^ CD11c^−^ lamina propria macrophages and found constitutive expression of *Epor* mRNA (Fig. 3A). *CD131* mRNA expression was increased by DSS-induced inflammation, while treatment with EPO or cibinetide inhibited this effect (Fig. 3B). In addition, we observed reduced p65 binding activity in sorted CD11b^+^ CD11c^−^ lamina propria macrophages from DSS-exposed mice treated with EPO or cibinetide as compared to cells from solvent treated littermates (Fig. 3C).

### Cibinetide acts on myeloid cells and inhibits the production of soluble disease mediators

**Figure 4:**
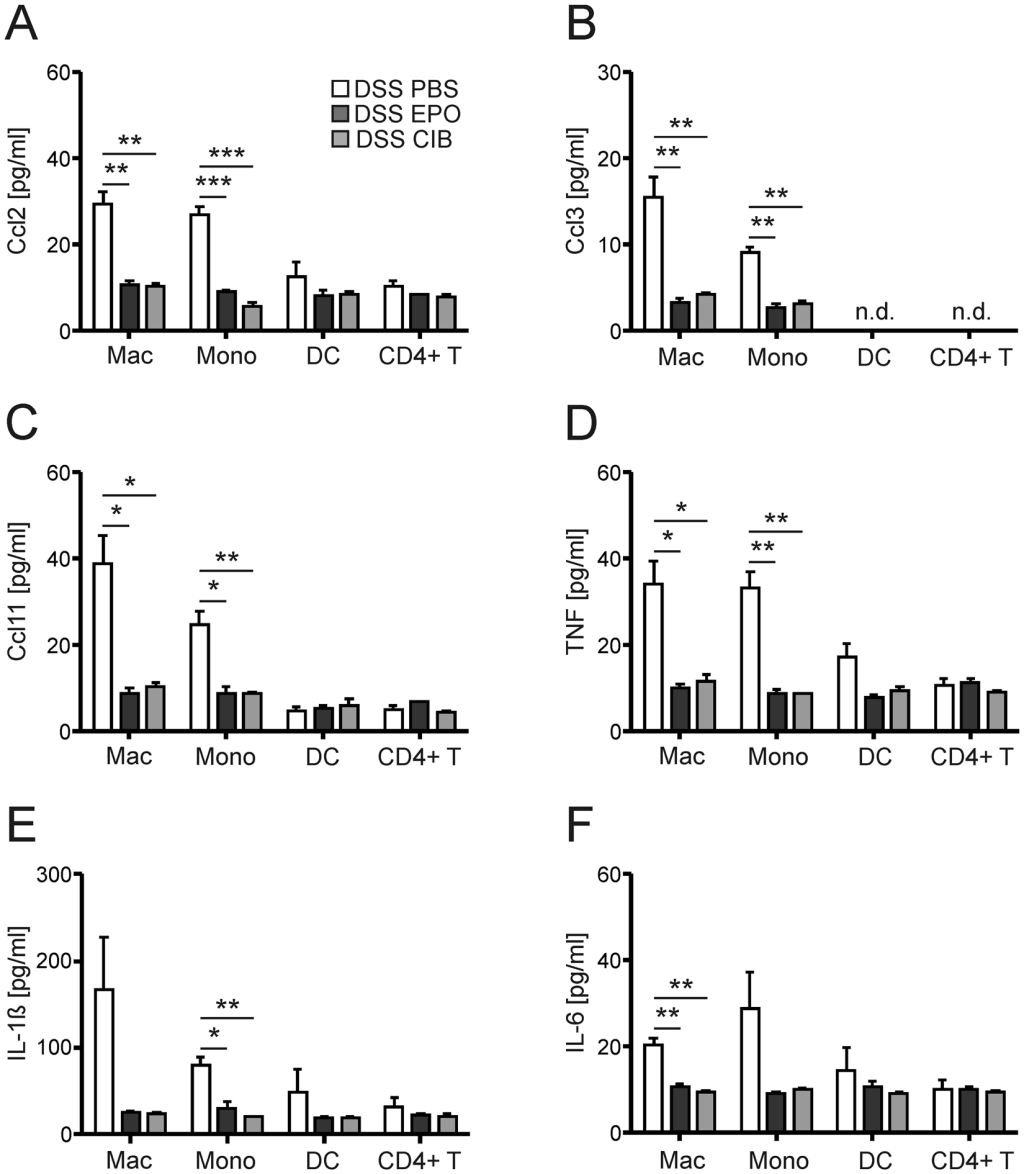
EPO and cibinetide inhibit chemokine and cytokine production by myeloid cells. The indicated immune cell populations were FAC-sorted from the lamina propria of DSS-exposed mice and cells were cultured for 24 hrs to allow for *ex vivo* mediator secretion. Concentrations of Ccl2 (**A**), Ccl3 (**B**), Ccl11 = Eotaxin; depicted in (**C**), TNF (**D**), IL-1ß (**E**) and IL-6 (**F**) were measured by specific ELISA kits. Data are from 3 to 4 individual mice and were compared using the unpaired student’s t-test. *p < 0.05, **p < 0.01, ***p < 0.001 as compared to DSS/PBS stimulation. n.d. denotes not detectable.

In line with these results, FAC-sorting of macrophages, monocytes, dendritic cells and CD4^+^ T cells from the lamina propria of DSS-exposed mice revealed that EPO and cibinetide both substantially reduced Ccl2, Ccl3, Ccl11, TNF, IL-1ß and IL-6 production by myeloid cells, but had only little effect on respective cytokine/chemokine expressions by CD4^+^ T cells (Fig. 4A–F). The observed clinical and histopathological improvement of colitis upon EPO or cibinetide treatment was also associated with reduced levels of nitrite, TNF, IL-1ß, IL-6, IL-12p70, IL-23, IFN-γ and IL-17A in supernatants of colonic organ cultures (Supplementary Fig. S4A–S4E). Corresponding results were found upon determination of cytokine and chemokine mRNA levels in colonic tissue samples from control and DSS mice demonstrating an inhibitory effect of EPO or cibinetide on the expression of several pro-inflammatory immune mediators (Supplementary Table 1).

### CD131 is necessary for macrophages to respond to cibinetide

**Figure 5:**
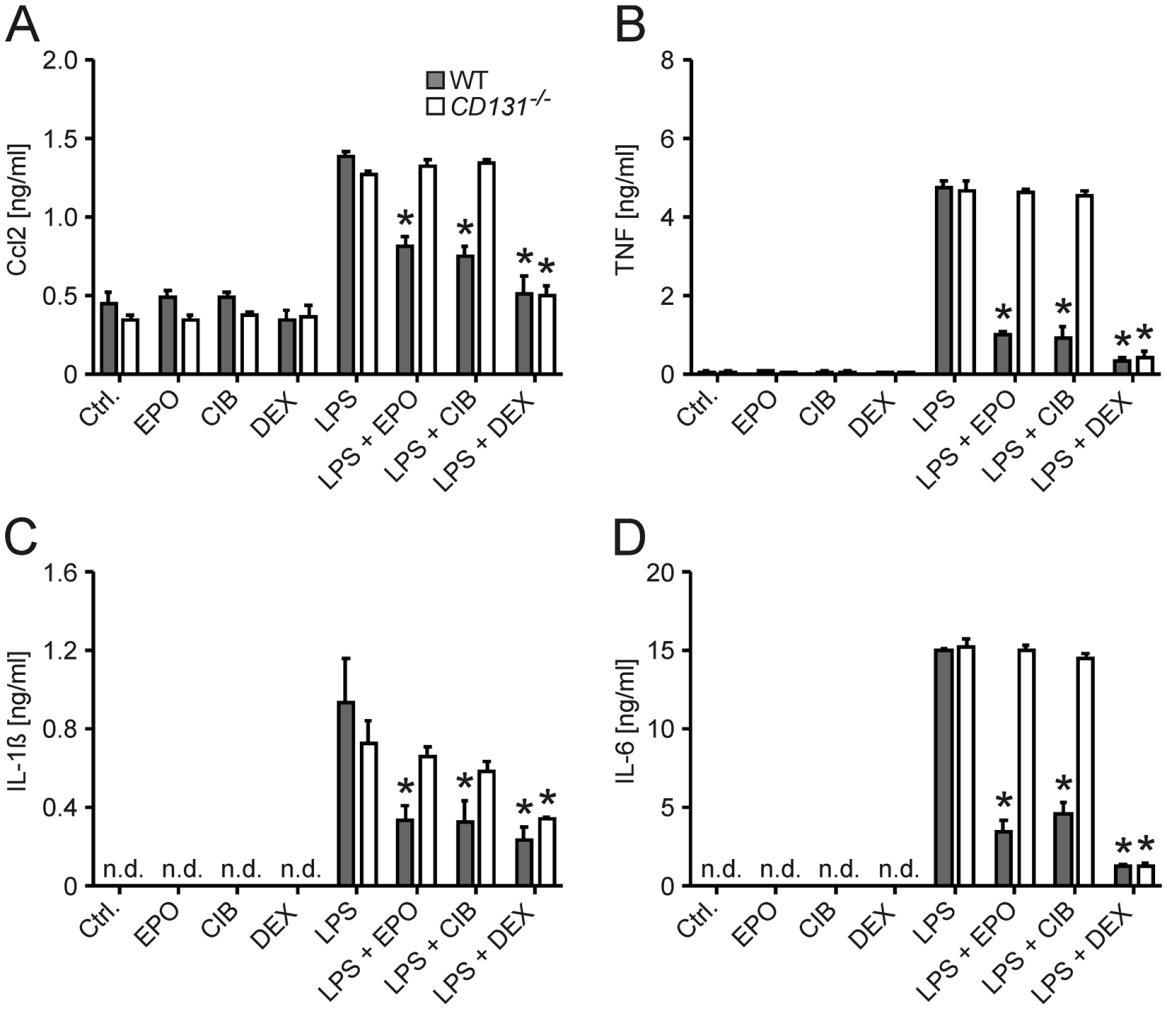
Cibinetide inhibits pro-inflammatory immune responses in BMDMs *in vitro* via CD131. BMDMs differentiated from *CD131* ^−/−^ mice or C57BL/6 N mice were pre-treated with PBS as solvent (Ctrl.), 2 pmol/mL rhu erythropoietin (EPO), 2 pmol/mL cibinetide (CIB) or 50 µg/ml dexamethasone (DEX) 30 min before the addition of LPS (200 ng/mL) or solvent. Supernatants were analyzed for concentrations of cytokines after 24 hours. Data from 4 independent experiments were compared by means of ANOVA using Bonferroni correction for multiple testing. Values are depicted as means ± S.E.M. and statistical significances between PBS and EPO, cibinetide or dexamethasone treatment, respectively, are indicated. Ccl2 (**A**), TNF (**B**), IL-1ß (**C**) and IL-6 (**D**) levels in supernatants are depicted. IL-1ß and IL-6 levels of solvent-treated control macrophages remained below the reported detection limits of the corresponding ELISA kits. *p < 0.05 as compared to LPS/solvent stimulation in the respective genotype group; n.d. denotes not detectable.

We next generated BMDMs from WT and *CD131* ^−/−^ mice and treated cells with EPO or cibinetide followed by stimulation with the TLR4 ligand LPS. We found that both, EPO and cibinetide, significantly reduced concentrations of Ccl2, TNF, IL-1ß and IL-6 in culture supernatants of LPS-stimulated WT BMDMs, but both compounds failed to affect cytokine concentrations in the absence of CD131 (Fig. 5A–D).

### Cibinetide acts on JAK2 and PI3 kinase and inhibits p65 activation

**Figure 6:**
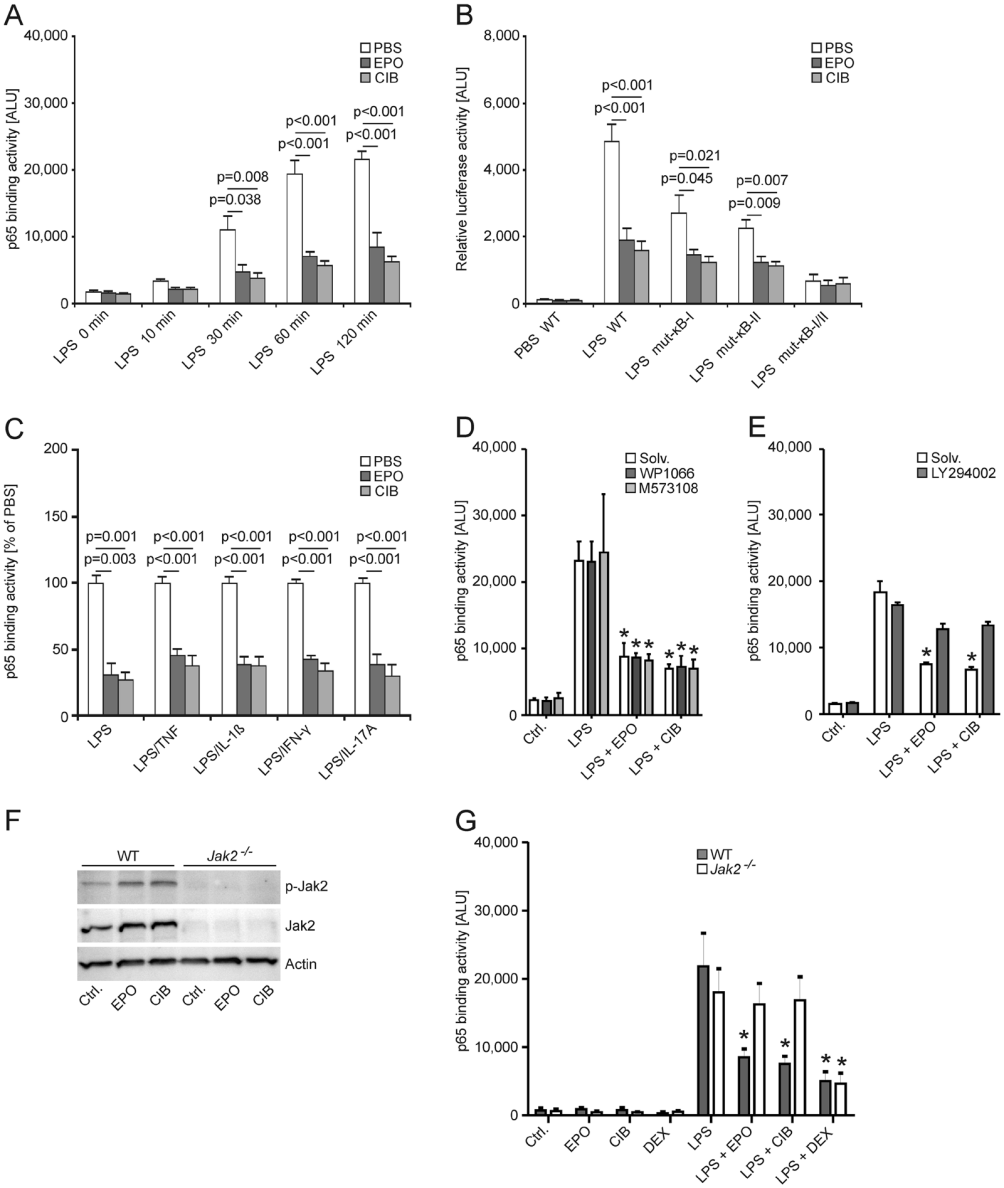
Cibinetide induces JAK2 phosphorylation and inhibits NF-κB p65 activation in BMDMs *in vitro*. (**A**) BMDMs were treated with PBS, 2 pmol/mL EPO, 2 pmol/mL cibinetide (CIB) and 200 ng/mL LPS. NF-κB p65 DNA binding activity in nuclear extracts was evaluated using a chemi-luminescent transcription factor assay at the indicated time points. Data of 3 independent experiments are expressed as arbitrary light units and shown as means ± S.E.M. and were compared using ANOVA. Statistically significant differences between LPS-stimulated cells pre-treated with either PBS, EPO or cibinetide are indicated. (**B**) BMDMs were transiently transfected with a full-length murine Nos2 promoter luciferase reporter or with constructs carrying site-specific mutations in one or both NF-κB binding sites (mut-κBI-Nos2-luc and mut-κBII-Nos2-luc or mut-κBI/II-Nos2-luc, respectively). Relative luciferase activity after pre-treatment with PBS, EPO or cibinetide and subsequent stimulation with LPS is shown. Data from 6 independent experiments were compared by ANOVA. (**C**) BMDMs were pre-treated as above and stimulated with LPS without and with co-stimulation with rmu TNF, IL-1ß, IFN-γ and IL-17A (10 ng/ml each). Nuclear NF-κB p65 binding activity was quantified. Data of 4 independent experiments are expressed as relative (to PBS-treatment) arbitrary light units and shown as means ± S.E.M. and were compared by ANOVA. Statistically significant differences between LPS-stimulated cells pre-treated with either PBS, EPO or cibinetide are indicated. (**D**,**E**) Following the addition of solvent, 10 µM WP1066, 200 µM M573108 or 50 µM LY294002, BMDMs were treated with PBS, EPO, cibinetide and LPS. Nuclear NF-κB p65 binding activity was measured. Data of 3 independent experiments are expressed as arbitrary light units and shown as means ± S.E.M. and were compared by means of ANOVA. *p < 0.001 as compared to the addition of LPS/solvent. (**F**) BMDMs were isolated from *Jak2* ^−/−^ mice and *Jak2* ^-+/+^ littermates. Cells were stimulated with PBS, EPO or cibinetide to verify the presence or absence of Jak2, respectively. (**G**) In independent experiments, BMDMs were stimulated with PBS, EPO, cibinetide (CIB) or 50 µg/ml dexamethasone (DEX). NF-κB p65 binding activity was quantified. *p < 0.05 as compared to LPS/solvent stimulation in the respective genotype group.

Moreover, pre-treatment of LPS-stimulated cells with EPO or cibinetide caused a significant reduction of p65 DNA binding activity in a time-dependent manner (Fig. 6A), which translated into reduced activation of the Nos2 promoter, because NF-κB is a central activator of Nos2 transcription. Notably, the effects of EPO and cibinetide were diminished in cells transfected with Nos2 promoter constructs carrying site-specific mutations in the NF-κB consensus sequence (Fig. 6B). The inhibitory effects of EPO and cibinetide on p65 activation were not limited to the TLR4 ligand LPS, but were also observed in BMDMs treated with PMA and ionomycin (data not shown) or in cells co-stimulated with LPS and TNF, IL-1ß, IFN-γ or IL-17A, respectively (Fig. 6C).

To determine whether the anti-inflammatory effects of EPO and cibinetide are dependent on JAK-STAT-dependent signaling or alternative pathways, we pre-incubated BMDMs with a panel of pharmacological inhibitors prior to the addition of EPO or cibinetide and stimulation with LPS, respectively. Inhibitors of STAT3 (WP1066) or STAT5 (M573108) did not interfere with EPO’s or cibinetide’s ability to de-activate p65 (Fig. 6D). Neither did we see an effect using inhibitors of the MAP kinases MEK or p38 (Data not shown). In contrast, pre-treatment with the PI3 kinase inhibitor LY294002 blunted the effects of EPO or cibinetide on p65 binding activity (Fig. 6E).

To corroborate these results, we generated BMDMs lacking JAK2 by Cre-lox technology (LysMCre-(LMC)-*Jak2* ^−/−^). While stimulated *LMC-Jak2* ^+/+^-BMDMs responded to EPO and cibinetide with JAK2-phosphorylation, no effect was found in *LMC-Jak2* ^*-/-*^BMDMs (Fig. 6F). Notably, pre-treatment with EPO or cibinetide failed to suppress p65 binding activity in *LMC-Jak2* ^*-/-*^BMDMs (Fig. 6G). In contrast, the glucocorticoid dexamethasone (DEX) did inhibit p65 binding activity independent of the presence or absence of JAK2.

### Cibinetide has little effect on cytokine production by T helper cells

As a range of T helper cell cytokines was significantly suppressed in the colon of DSS-exposed mice treated with EPO or cibinetide (Supplementary Table 1), we tested whether EPO or cibinetide may also act on isolated CD4^+^ T cells stimulated *in vitro* with PMA/ionomycin or with conditioned media (CM) of BMDMs treated with PMA/ionomycin and PBS (as solvent), EPO or cibinetide. We found a fairly small and non-significant inhibitory effect of EPO and cibinetide on IL-17A mRNA expression in isolated CD4^+^ cells stimulated with PMA/ionomycin (Supplementary Fig. S5C). In contrast, conditioned media of BMDMs treated with PMA/ionomycin and PBS (as solvent), EPO or cibinetide had differential effects on the mRNA expression of TNF, IFN-γ, IL-17A, IL-4 and IL-6 dependent on whether or not conditioning of BMDMs included EPO or cibinetide (Supplementary Fig. S5A–S5E), further evidence that EPO and cibinetide act primarily on myeloid cells.

## Discussion

The results of this study demonstrate that cibinetide exerts broad and potent anti-inflammatory effects on myeloid cells. *In vitro*, cibinetide significantly reduced the production of several macrophage-derived inflammatory mediators in response to LPS stimulation. *In vivo*, the proportion of TNF^+^ monocytes and macrophages in the colonic lamina propria was reduced after treatment of DSS-exposed mice with cibinetide or EPO. The observation that *in vivo* cibinetide treatment dampens the *ex vivo* secretion of Ccl2, Ccl3, Cxcl1 and Ccl11 by FAC-sorted monocytes and macrophages further suggests that the recruitment of inflammatory cells to the colonic lamina propria is dampened by activation of the IRR of myeloid cells. Since cibinetide has no effect on erythropoiesis, it appears unlikely that the compound may influence the ratio of erythropoiesis/myelopoiesis. In fact, the proportion of Ter119^+^ vs. CD11b^+^ cells in the bone marrow was not affected by cibinetide. Moreover, IL-10 and TGF-ß mRNA levels in the colon were dampened by cibinetide or EPO treatment in the same manner as were pro-inflammatory cytokines and chemokines. We therefore propose a direct anti-inflammatory effect of cibinetide downstream of the IRR. The *in vitro* results obtained with JAK2 deficient BMDMs and with selective kinase inhibitors support this concept and further suggest that JAK2 and PI3K transduce this anti-inflammatory signal to the nucleus. Although EPO is known to influence several signal transduction cascades, we found that in LPS- or cytokine-stimulated macrophages, EPO and cibinetide primarily targeted the NF-κB subunit p65 activation pathway following JAK2 phosphorylation. Since NF-κB is one of the crucial transcription factors in the initiation and amplification of the inflammatory cascade, cibinetide and EPO both influence a key event of macrophage activation.

Inflammatory mediators such as TNF and IL-1ß auto-regulate their own expression via a positive amplification loop involving NF-κB. Moreover, NF-κB coordinates the expression of a variety of other inflammatory genes, including adhesion molecules and additional transcription factors. NF-κB has further been shown to be centrally involved in the pathophysiology of human inflammatory bowel disease (IBD) and experimental colitis. The increased risk of Crohn’s disease observed in individuals with pathogenic nucleotide-binding oligomerization domain (NOD)−2 polymorphisms is believed to result from increased NF-κB binding activity in the colon and enhanced secretion of IL-1ß^29^. Furthermore, lamina propria macrophages in IBD patients have increased NF-κB binding activity along with increased production of TNF, IL-1ß and IL-6^30,31^. Experimental treatment strategies which specifically target p65 functions ameliorate disease severity in different models of murine colitis^32–36^.

Inhibition of NF-κB activation is thus a direct and promising intervention for the treatment of distinct forms of colitis. Cibinetide may have the capacity to beneficially interfere with the autocrine and paracrine stimulation of cytokine production by macrophages and other immune cells within inflamed microenvironments. Of relevance, medications used in the treatment of IBD such as glucocorticoids, sulfasalazine, mesalazine and leflunomide act, at least in part, via inhibition of NF-κB activity^37^. Consequently, the addition of cibinetide to established medications may help to accomplish dose reductions, thus limiting toxic side effects associated with current therapeutic regimens. Moreover, cibinetide may be especially valuable for patients who are primarily resistant to conventional therapies or experience tachyphylaxis^38,39^. An additional benefit is that cibinetide lacks side effects that are ascribed to the erythropoiesis inducing activity of EPO, such as hyperviscosity of the blood and an increased risk for thromboembolic events^40^.

In these studies we have demonstrated profound anti-inflammatory and disease modifying activity of cibinetide in the setting of DSS-induced colitis without evidence of unintended erythropoietic effects. The results of our studies are encouraging to initiate a randomized controlled trial to study the therapeutic potential of cibinetide in human IBD.

## Materials and Methods

### Compounds

Cibinetide (CIB), also known as ARA 290 or pyroglutamate helix B surface peptide (pHBSP), was obtained from Araim Pharmaceuticals (Tarrytown, NY, USA). Recombinant human (rhu) EPO theta (Eporatio®) was purchased from Ratiopharm (Vienna, Austria). Both compounds were diluted in phosphate-buffer saline (PBS).

### Animals

Conditional deletion of *Jak2* in lysozyme M positive cells was performed by crossing *Jak2f/f* mice, kindly provided by K.U. Wagner (Eppley Institute for Research in Cancer and Allied Diseases, University of Nebraska, Omaha, NE), with *LysMCre* transgenic mice^41^ and by further breeding to generate *Jak2f/f*;*LysMCre* (*LMC-Jak2* ^−/−^) animals. *Jak2* ^−/−^ animals and *Jak2* ^+/+^ littermates were used on a mixed C57BL/6N-Sv129 background^42,43^. *CD131* ^−/−^ mice were generated and used as described^44,45^.

### Establishment of DSS-Colitis

DSS-colitis was induced in male, pathogen-free, 8–12 weeks old C57BL/6N mice (Charles River Laboratories) following a reported protocol with slight modifications^46^. Briefly, 3% dextran sulfate sodium (DSS; MW 36,000–50,000; obtained from MP Biomedicals) was added to the drinking water with *ad libitum* availability to mice. Animals were treated with DSS or water for 7 consecutive days, followed by 7 days of water. Starting on day 8 after the initiation of DSS treatment, mice were daily administered 1.5 pmol/g body weight rhuEPO or cibinetide, while control mice received PBS. All animal experiments were performed according to the guidelines of the Medical University of Innsbruck based on the Austrian Animal Testing Act of 1988. All animal experiment protocols were approved by the ‘MUI Animal ethics committee’ and the Austrian Ministry for Science and Education (approval no. BMWF-66.011/0020-II/3b/2012).

### Cell Isolation and Culture

Age and sex matched mice were sacrificed, bone marrow cells were recovered from tibiae and femora and erythrocytes were lysed. BMDMs were initially cultured on non-tissue culture dishes using 100 ng/ml recombinant murine (rmu) M-CSF (Prepotech). BMDMs used in assays were between 6 d and 8 d in culture and were grown overnight on tissue culture 6-well plates prior use.

CD4^+^ T cells were isolated from spleen by positive selection using a specific MACS system (Miltenyi Biotec) according to the manufacturer’s instructions.

Cells were incubated with 2 pmol/mL rhuEPO or cibinetide diluted in PBS or PBS alone. After another 30 min, cells were stimulated with 200 ng/mL LPS (lipopolysaccharide from Escherichia coli 055:B5), 100 ng/ml PMA (phorbol-12-myristate-13acetate), 500 ng/ml ionomycin (Sigma) and/or 10 ng/mL rmu TNF, IL-1ß, IFN-γ or IL-17A (R&D) while controls were treated with PBS. Thereafter, supernatants were harvested and cells were subjected to RNA or protein preparation. The use of either rhuEPO theta (Eporatio®; Ratiopharm) or of rmuEPO (R&D) yielded very similar results. Thus, rhuEPO was used for subsequent experiments. Where indicated, 100 µM AG490 (JAK2 inhibitor), 10 µM WP1066 (STAT3 inhibitor), 200 µM M573108 (STAT5 inhibitor), 50 µM PD98059 (MEK inhibitor), 500 µM SB203540 (p38 MAPK inhibitor; all obtained from Merck), 50 µg/ml CAPE (NF-κB inhibitor; from Marlingen), 50 µM LY294002 (PI3K inhibitor; Merck) or 50 µg/ml dexamethasone (Sigma) were used. PBS, DMSO (Sigma) or SB202474 (negative control for SB203540; Merck) served as appropriate controls.

Colonic lamina propria cells were isolated from the large intestine as described previously^47,48^. In brief, the colon was opened longitudinally, cut into small pieces and washed extensively in PBS to remove feces and debris. Following incubation in HBSS containing 5% FCS, 1.5 mg/ml collagenase VIII and 40 µg/ml DNase I (Sigma), suspensions of lamina propria cells were directly stained for flow cytometry or cell sorting. Individual cell populations were sorted as detailed in the Supplementary information section. Purity of isolated cell populations was at least 98% as determined by FACS analysis.

For human mononuclear cell cultures, 1–3 × 10^5^/ml mononuclear cells (MNC) isolated by lymphoprep from peripheral blood of healthy donors were dissolved in IMDM/5% FCS, counted and mixed with methylcellulose (Methocult^TM^ GF^+^ H4535 without EPO, StemCell Technologies, Germany) and either solvent or increasing concentrations of EPO (0.4 or 1.2 pmol/mL rhuEPO) or CIB (0.4, 1.2 or 12 pmol/mL cibinetide). Cells were cultured for 14 days and BFU-E were identified according to their morphology with an inverted microscope and a 100 mm culture dish marked with the scoring grid. For analysis of hemoglobin content and mRNA expression, colonies were picked according to their morphology, pooled in PBS and frozen for later analyses. All methods were carried out in accordance with the guidelines and regulations of the Medical University Innsbruck. All experimental protocols were approved by the bioethical committee ‘Ethikkommission’ of the Medical University Innsbruck. Written informed consent was obtained from all subjects.

### Flow Cytometry

Cell suspensions were stained in PBS supplemented with sterile 1% FBS and 0.5% BSA using the antibodies listed in the Supplementary information section.

### RNA extraction and quantitative real-time PCR

Preparation of total RNA and quantification of mRNA expression by quantitative Taqman RT-PCR following reverse transcription was performed exactly as described^16^. For colon samples, the Dynabeads® mRNA kit was used (Ambion).

### Protein extraction and Western blot analysis

Protein extraction and Western blotting were performed exactly as described^49^. NF-κB p65 binding activity in nuclear extracts was assessed using a chemi-luminescent assay kit according to the manufacturer’s instructions (Pierce).

### Transient Transfections

The full-length Nos2 promoter (WT-Nos2-luc), cloned into the pGL2 basic luciferase reporter gene vector, as well as Nos2-luciferase reporter constructs bearing point mutations at the NF-κB consensus sequences of either the proximal region I (mut-κBI-Nos2-luc), the distal region II (mut-κBII-Nos2-luc), or at both sites (mut-κBI/II-Nos-luc) were used^50,51^. Nos2 promoter activity was determined by the Dual Luciferase system (Promega) according to the manufacturer’s instructions.

### Detection of cytokines, reactive species and hemoglobin

Determination of cytokines in culture supernatants was performed with specific ELISA kits (from R&D). Hemoglobin content was measured in a spectrophotometer at a wave length of 660 nm based on the heme-catalyzed oxidation of tetramethylbenzidine by H_2_O_2_ using serial dilutions of purified human hemoglobin (Sigma) as standard^52^. Values were normalized for total protein of bone marrow cells as determined by BCA (Pierce) or for dry weight of fecal content.

### Histopathology

Histological examinations of tissues from *in vivo* infection and colitis experiments, respectively, were performed on formalin-fixed tissue sections stained with hematoxylin and eosin (HE) according to a standard protocol. The degree of colonic inflammation on microscopic sections was graded semi-quantitatively as described^28^. Histological scoring was performed by an experienced pathologist blinded to study design and sample identity.

### Statistical analysis

Statistical analyses were carried out using SPSS using unpaired two-tailed student’s t-tests or by Mann-Whitney U test to assess data where only two groups existed. Analysis of variance combined with Bonferroni correction or Kruskall-Wallis test, as appropriate, was used for all other experiments. Survival was compared by log-rank test. *P* values less than 0.05 were considered significant.

## Electronic supplementary material

Supplementary Information
