# Supplementary material for: Cibinetide dampens innate immune cell functions thus ameliorating the course of experimental colitis
Source: Sci Rep. 2017 Oct 12;7:13012. doi: 10.1038/s41598-017-13046-3 (PMC5638901; doi:10.1038/s41598-017-13046-3)
Supplement: Supplementary file 1 — Supplementary Information [file 41598_2017_13046_MOESM1_ESM.pdf]

Cibinetide dampens innate immune cell functions thus ameliorating the course of experimental colitis

Manfred Nairz[1-3,*,#], David Haschka[1,#], Stefanie Dichtl[1,#], Thomas Sonnweber[1], Andrea Schroll[1], Malte Aßhoff[1], John E. Mindur[2,3], Patrizia L. Moser[4], Dominik Wolf[5], Filip K. Swirski[2,3], Igor Theurl[1], Anthony Cerami[6], Michael Brines[6], Günter Weiss[1]

Supplementary Methods.

List of antibodies used.

The following monoclonal antibodies and gating strategies were used for flow cytometric analysis:

anti-Ly-6C (clone AL-21, BD Biosciences, San Jose, CA, USA), anti-CD45.2 (clone 104, BD Biosciences), anti-CD3e (clone 145-2C11, ebioscience, San Diego, CA, USA), anti-CD90.2 (clone 53-2.1, BD Biosciences), anti-CD19 (clone 6D5, Biolegend, San Diego, CA, USA), anti-B220 (clone RA3-6B2, BD Biosciences), anti-MHCII (clone AF6-120.1, BD Biosciences), anti-F4/80 (clone BM8, Biolegend), anti-CD49b (clone DX5, BD Biosciences), anti-NK1.1 (clone PK136, BD Biosciences), anti-Ly-6G (clone 1A8, BD Biosciences), anti-Gr-1 (clone RB6-8C5, BD Biosciences), anti-CD11b (clone M1/70, BD Biosciences), anti-CD11c (clone HL3, BD Biosciences), anti-Ter119 (clone Ter-119, BD Biosciences), anti-CD115 (clone AFS98, ebioscience), anti-SiglecF (clone E50-2440, BD Biosciences), anti-IgE (clone 23G3, ebioscience), anti-FcεRI (clone MAR-1, ebioscience),
anti-TNF (clone MP6-XT22, Biolegend), anti-Nos2 (clone CXNFT, Biolegend), anti-IFN-γ (clone XMG1.2, Biolegend), anti-IL17A (clone TC11-18H10.1, Biolegend), anti-IL10 (clone JES5-16E3, Biolegend), anti-CD25 (clone PC61, Biolegend), anti-CD64 (clone X54-5/7.1, Biolegend), anti-CX3CR1 (clone SA011F11, Biolegend), anti-IgA (clone C10-3, BD Biosciences);

Cells were first gated using FSC/SSC characteristics, and doublets were sequentially excluded by comparing FSC- and SSC-height and -area signals. Data were acquired on a Gallios (Becton Coulter) or LSRII (BD Biosciences) flow cytometer and analyzed with FlowJo v8.8.6 (Tree Star, Inc., Ashland, USA). For cell sorting, monocytes, macrophages, DCs and T cells from colonic lamina propria homogenates were isolated by FACS with a FACS Aria II cell sorter.

Supplementary Data.

Supplementary Figure S1. Lack of erythropoietic activity of CIB.

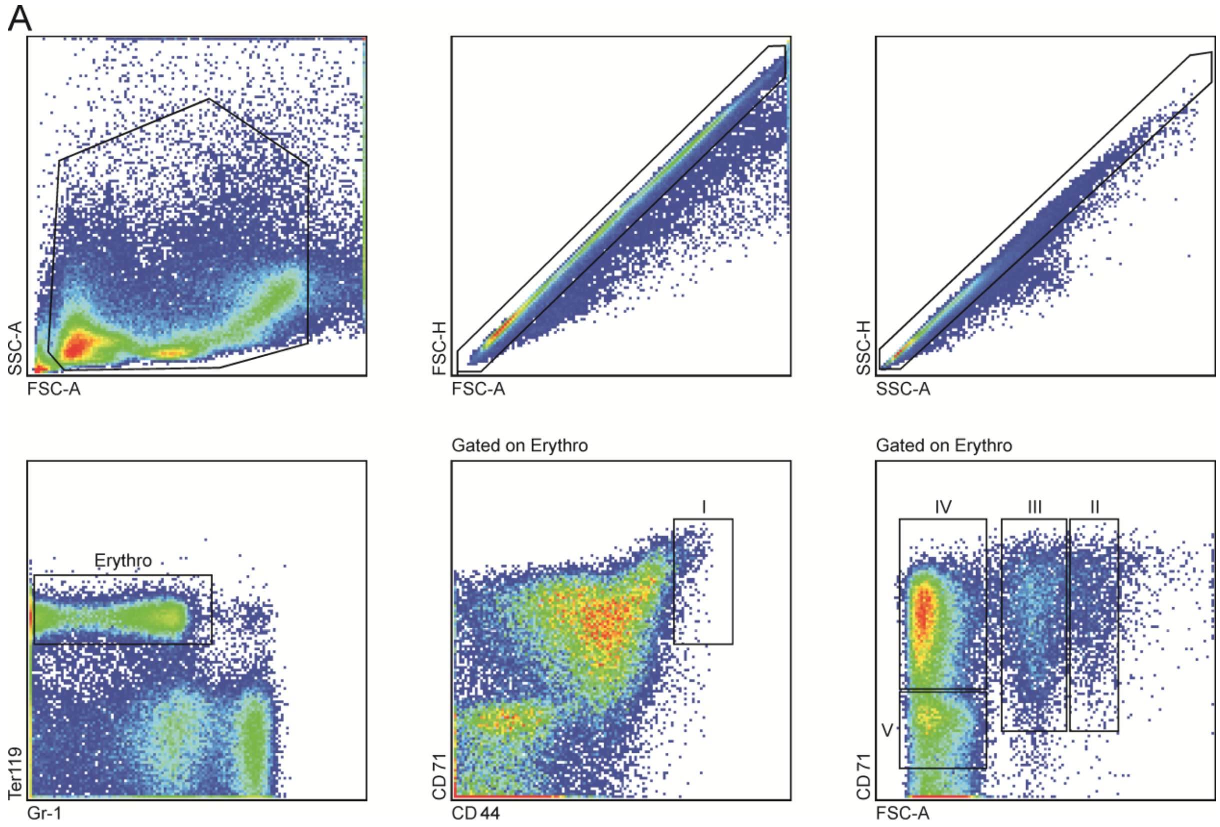

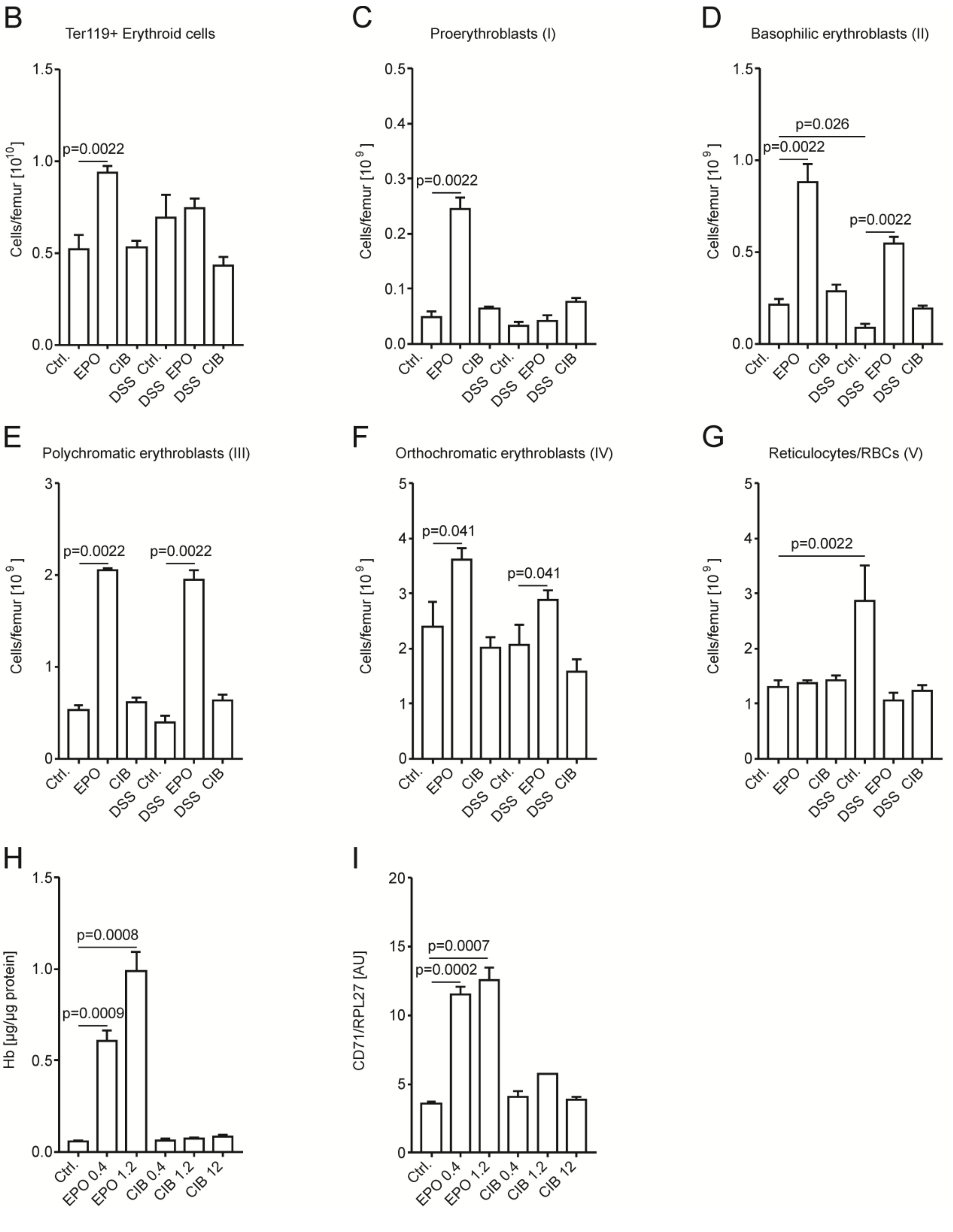

(A) Gating strategy to characterize erythropoiesis. Bone marrow cells from 1 femur and 1 tibia per mouse were analyzed by flow cytometry.

(B-G) Erythroid differentiation in mice treated with erythropoietin (EPO), cibinetide (CIB) or PBS (Ctrl.; used as solvent) and exposed to DSS.

(B) Erythroid cells were identified as $Ter119^{+}$, $CD45^{-}$, $Gr\text{-}1^{-}$ cells. FSC characteristics, CD44 and CD71 were used to follow erythroid differentiation: (C) $CD44^{high}$, $CD71^{high}$ (proerythroblasts), (D) $CD71^{high}$ $FSC\text{-}A^{high}$ (basophilic erythroblasts), (E) $CD71^{high}$ $FSC\text{-}A^{int.}$ (polychromatic erythroblasts), (F) $CD71^{high}$ $FSC\text{-}A^{low}$ (orthochromatic erythroblasts) and (G) $CD71^{low}$ $FSC\text{-}A^{low}$ (reticulocytes and erythrocytes) populations. n = 5-12 per group.

(H) PBS as solvent (Ctrl.), 0.4 or 1.2 pmol/mL rhuEPO or 0.4, 1.2 or 12 pmol/mL CIB were added to human mononuclear cell cultures. Hemoglobin (Hb) content relative to total protein content was measured in a spectrophotometer. Data representing 3 healthy subjects were compared by Kruskal-Wallis testing.

(I) CD71/TFR1 mRNA expression relative to the housekeeping gene RSP27 was assessed as a surrogate for erythroid differentiation.

Supplementary Figure S2. EPO and CIB inhibit myeloid Nos2 expression.

A

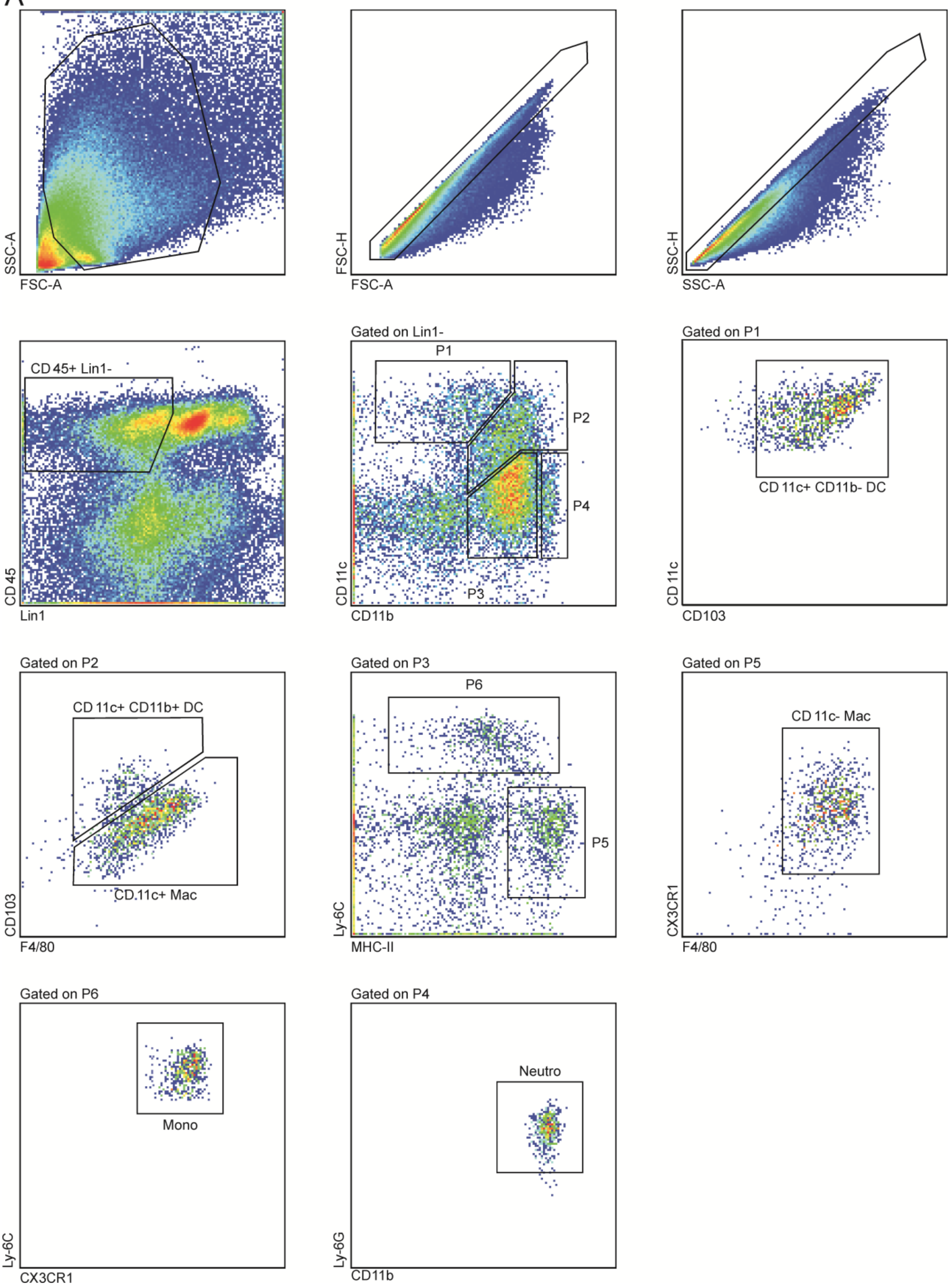

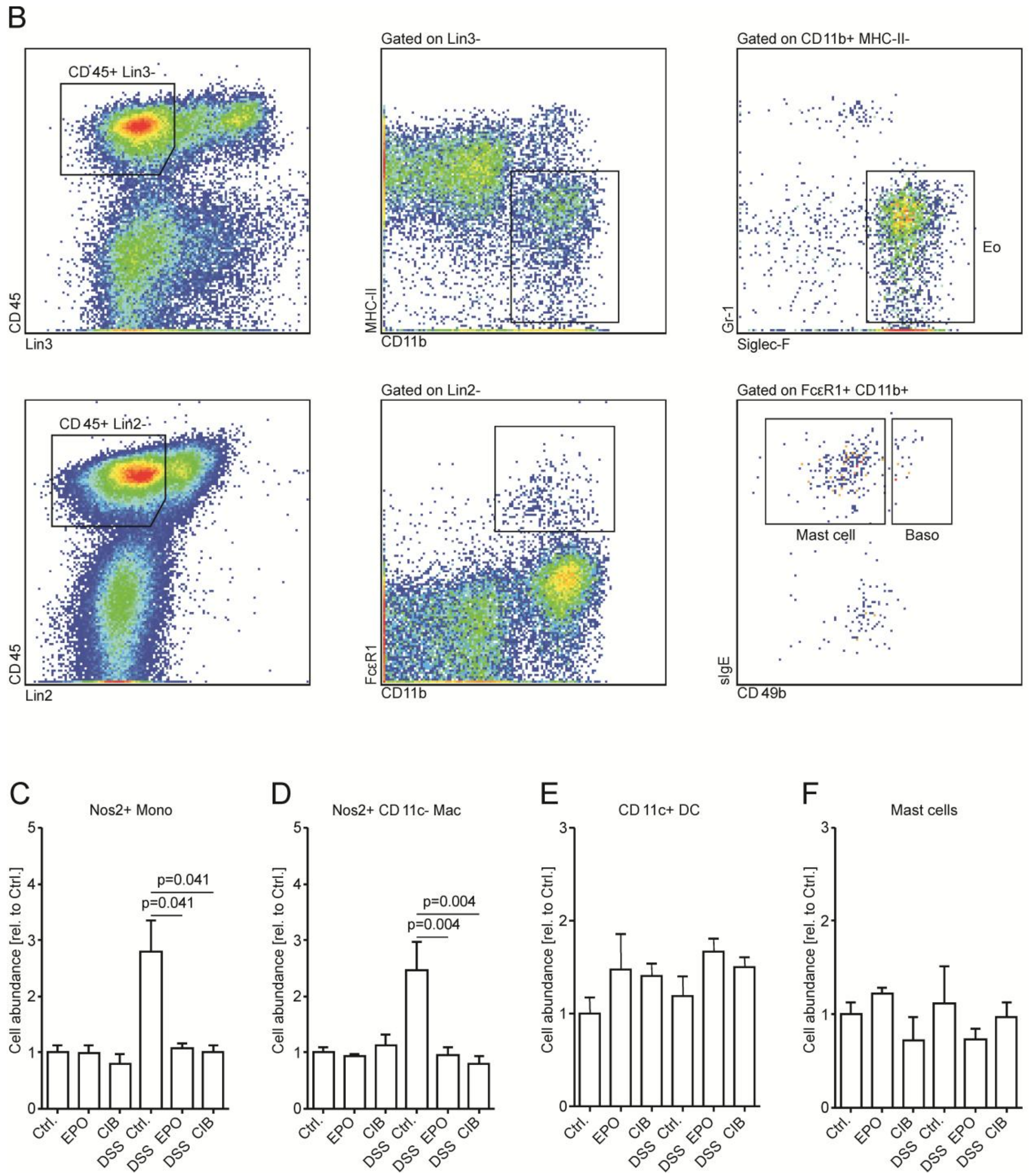

(A, B) Gating of lamina propria myeloid cells. (A) CD11b$^-$ dendritic cells (DC) were identified as CD45$^+$, Lin1$^-$ (Lin1 = B220, CD3, CD19, CD90.2, NK1.1, FcεR1, SiglecF), CD11c$^+$, CD11b$^-$, CD103$^+$, F4/80^{low}, MHCII^{high} (P1). CD11b$^+$ DC were identified as CD45$^+$, Lin1$^-$, CD11c$^+$, CD11b$^+$, CD103$^+$, F4/80^{low}, MHCII^{high} (in P2). CD11c$^+$ macrophages (Mac) were identified as CD45$^+$, Lin1$^-$, CD11c$^+$, CD11b$^+$, CD103$^-$, F4/80^{high}, CX3CR1$^+$, MHCII^{high} (in P2). CD11c$^-$ macrophages were identified as CD45$^+$, Lin1$^-$, CD11c$^-$, CD11b$^+$, CD103$^-$, F4/80^{high}, CX3CR1$^+$, MHCII^{high} (P5).

Monocytes were identified as $CD45^+$, $Lin1^−$, $CD11b^+$, F4/80^{low}, $MHCII^{low}$, $CD115^+$ cells (P6). Neutrophils were identified as $CD45^+$, $Lin1^−$, $CD11b^+$, F4/80^-, $MHCII^{low}$, SSC^{high}, Ly-$6G^{high}$ cells (P4).

(B) Eosinophils were identified as $CD45^+$, $Lin3^−$ (Lin3 = B220, CD3, CD11c, CD19, CD90.2, NK1.1), $CD11b^+$, $MHCII^{low}$, SSC^{high}, Gr-1^-, $CX3CR1^-$, $SiglecF^{high}$ cells.

Mast cells were identified as $CD45^+$, $Lin2^−$ (Lin2 = CD3, CD4, CD11c, CD19, CD90.2, NK1.1), $CD11b^+$, $MHCII^{low}$, $Fc\varepsilon R1^+$, $CD49b^-$, surface IgE^{high} cells, whereas basophils were $CD49b^+$.

(C, D) Effect of EPO and CIB on Nos2 expression in Ly-$6C^{high}$ monocytes (C) and $CD11c^-$ macrophages (D). Numbers represent cell abundance relative to PBS-injected controls kept on drinking water (Ctrl.). To assess Nos2 expression by flow cytometry, single cell suspensions were re-stimulated with PMA and ionomycin. Cells were permeabilized for intracellular staining. Data were compared by Kruskal-Wallis testing. n = 5-12 per group.

(E, F) EPO and CIB do not alter the relative numbers of $CD11c^+$ DCs (E) or mast cells (F) in the lamina propria as quantified by flow cytometry. n = 5-12 per group.

Supplementary Figure S3. No effect of EPO or CIB on IL-10$^+$ CD4$^+$ T cells in vivo.

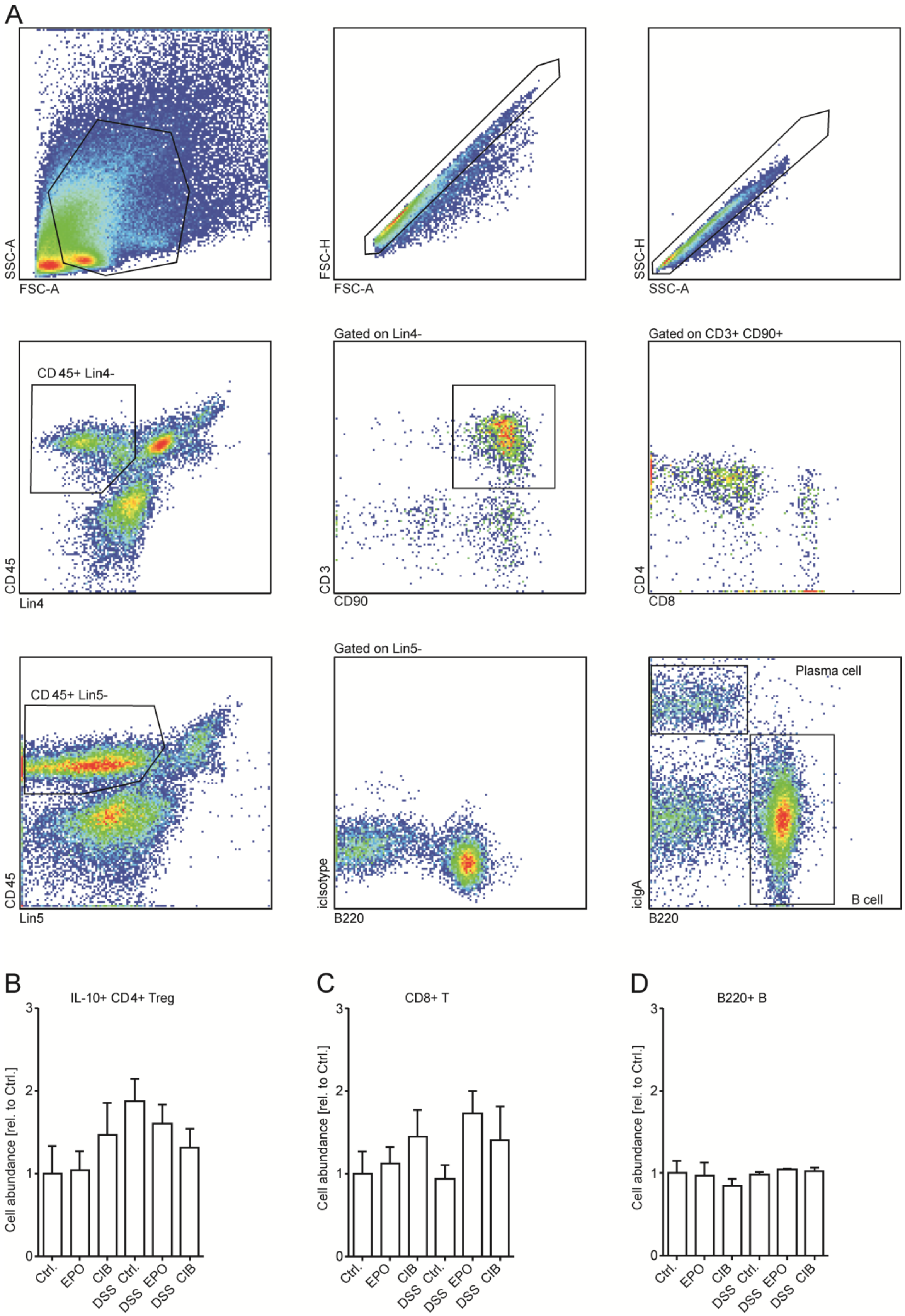

(A) Gating of lamina propria T and B cells. T cells were identified as $CD45^+$, $Lin4^-$ (Lin4 = B220, CD19, CD11b, CD11c), $NK1.1^-$, $CD3^+$, $CD90^+$, subdivided into $CD4^+$ and $CD8^+$.

B cells were identified as $CD45^+$, $Lin5^-$ (Lin5 = CD3, CD11b, CD11c, CD90.2, NK1.1, FcεR1, SiglecF), $B220^+$, $CD19^+$, $MHCII^{high}$, intracellular IgA^-, plasma cells were intracellular IgA^+.

To assess cytokine expression by flow cytometry, single cell suspensions were re-stimulated with PMA and ionomycin in the presence of Golgi blockage. Cells were permeabilized for intracellular staining. Data were compared by Kruskal-Wallis testing. n = 5-12 per group

(B-D) EPO and CIB do not affect the abundance of IL-10^+ $CD4^+$ T cells (B), $CD8^+$ T cells (C) or $B220^+$ B cells (D). n = 5-12 per group.

Supplementary Figure S4. Reduced cytokine production in colonic organ cultures.

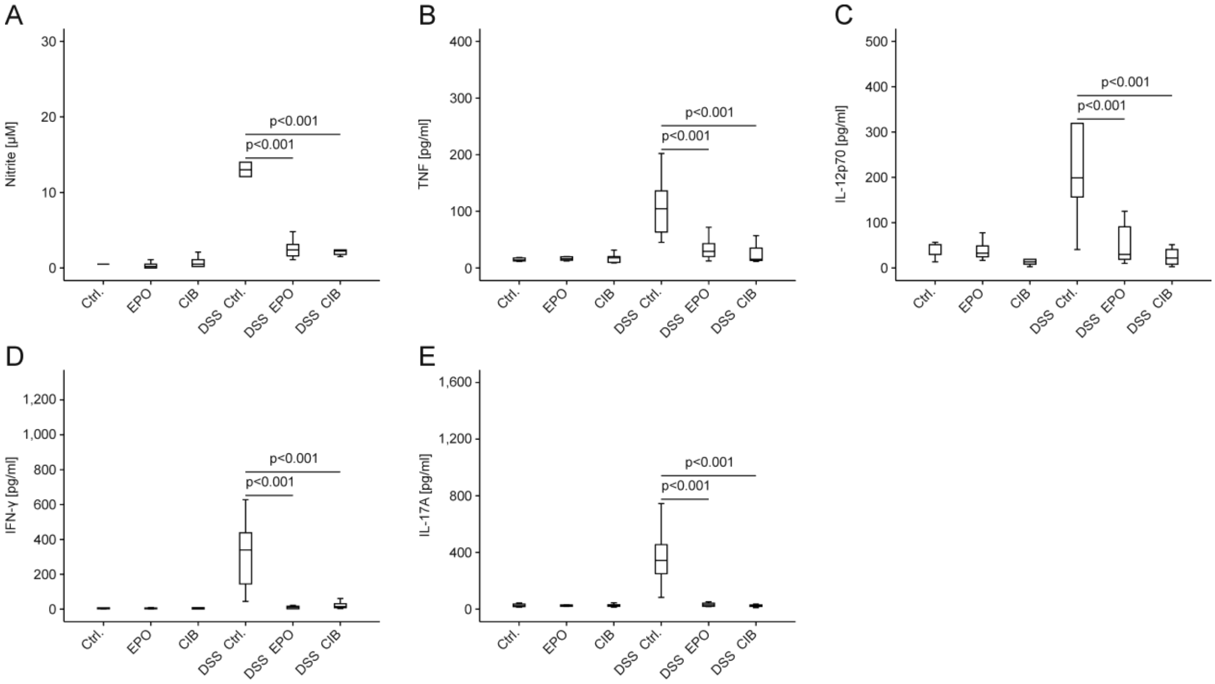

Colon samples were explanted from control and DSS-exposed mice described in the legend to figure 1A on d14 and cultured for 24 hours to allow for cytokine production (n = 5-12 per group).

Nitrite (A) and cytokine (B-E) concentrations were determined by the Griess reaction and ELISA, respectively. Data from 2 independent experiments were compared by means of ANOVA. Values are depicted as lower quartile, median and upper quartile (boxes), and minimum/maximum ranges and statistical significances are indicated.

Supplementary Figure S5. No effect of EPO or CIB on the activation of CD4^{+} T cells in vitro.

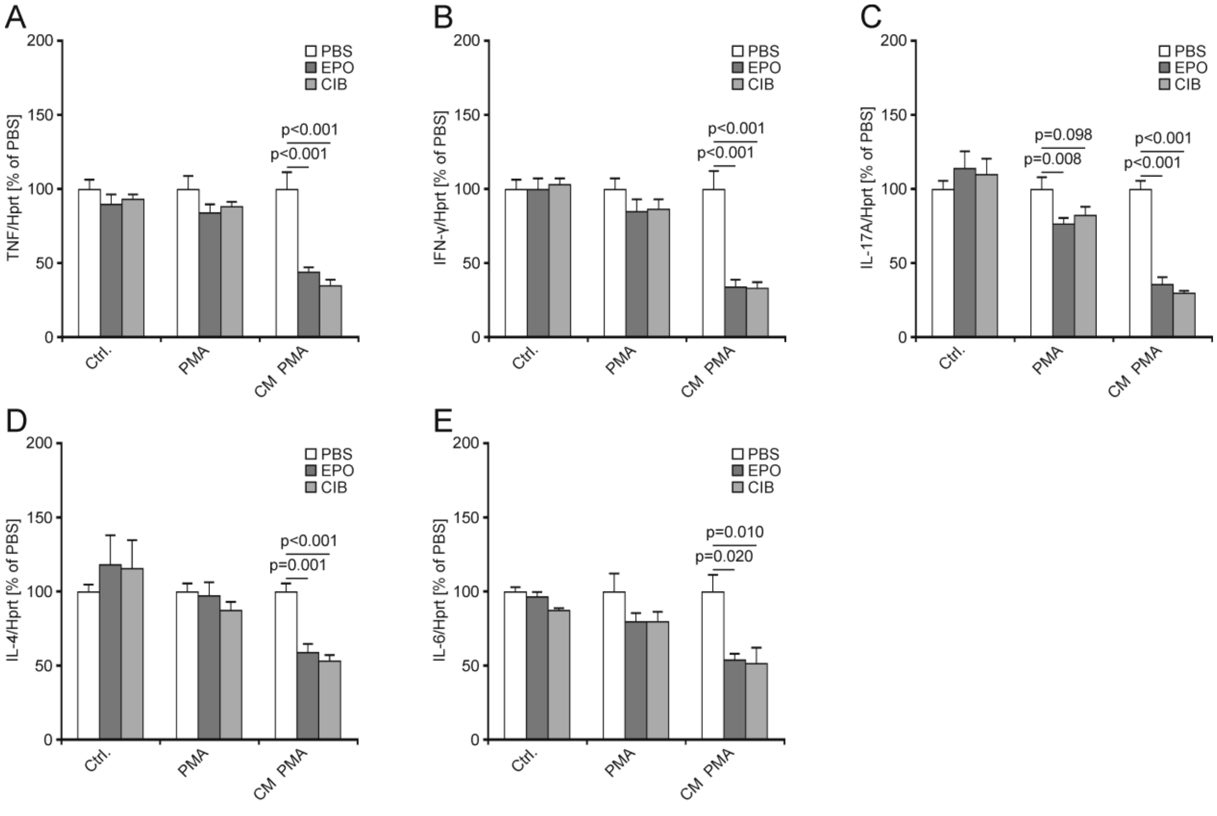

(A-E) CD4^{+} T cells were isolated from the spleen of naïve C57BL/6N mice and stimulated with PBS, EPO or CIB. Where appropriate, 100 ng/ml PMA and 500 ng/ml ionomycin or conditioned media from PMA/ionomycin-stimulated BMDMs (treated with PBS, EPO or CIB for 24 hours) were added. TNF (A), IFN-γ (B), IL-17A (C), IL-4 (D) and IL-6 (E) mRNA expression was studied by qRT-PCR.

Supplementary Table 1. CIB affects the colonic expression of a range of immune response genes in the setting of DSS-colitis.

Colon samples of DSS-exposed mice treated with PBS (Ctrl.; used as solvent), rhu erythropoietin (EPO) or cibinetide (CIB) were analyzed by qRT-CPR on d14. Expression relative to the house keeping gene Hprt was quantified. Data are shown as means ± S.E.M. for 5-12 mice per group. Results were compared using ANOVA. Statistical significant differences between DSS-mice treated with EPO or CIB in comparison to PBS treatment (Ctrl.) are indicated.

Supplementary Table 1. CIB affects the colonic expression of a range of immune response genes in the setting of DSS-colitis.

Treatment	Ctrl.	EPO	CIB	DSS + Ctrl.	DSS + EPO	DSS + CIB
Gene of interest						
Ccl2	2.91± 1.40	1.68 ± 0.89	3.08 ± 1.26	40.82 ± 15.73	0.58 ± 0.22 [b]	2.88 ± 1.44
Ccl3	2.93 ± 0.87	2.17 ± 0.64	2.86 ± 1.21	45.63 ± 13.40	1.18 ± 0.44 [b]	2.18 ± 0.66 [a]
Ccl11	2.29 ± 0.86	3.43 ± 1.62	2.90 ± 1.04	377.80 ± 173.70	0.42 ± 0.17 [c]	3.10 ± 1.74 [a]
Cxcl1	1.77 ± 0.70	2.93 ± 1.50	2.92 ± 1.76	22.46 ± 8.10	0.53 ± 0.15 [a]	3.49 ± 2.05
Cxcl2	2.67 ± 0.92	4.14 ± 1.66	3.25 ± 0.69	30.19 ± 13.59	1.18 ± 0.30	1.19 ± 0.40 [a]
Mcp1	4.31 ± 1.84	4.77 ± 2.46	3.64 ± 1.56	83.08 ± 26.76	0.63 ± 0.24 [b]	5.40 ± 2.37
Nos2	0.67 ± 0.18	0.53 ± 0.13	0.42 ± 0.17	352.35 ± 66.91	4.53 ± 1.20 [c]	2.92 ± 0.87 [c]
TNF	17.91 ± 3.39	20.42 ± 1.78	7.93 ± 2.56	182.59 ± 29.33	13.19 ± 3.08 [c]	12.87 ± 5.63 [c]
IL-1ß	0.22 ± 0.07	0.22 ± 0.04	0.19 ± 0.04	64.62 ± 19.60	4.90 ± 1.34 [c]	2.35 ± 0.79 [c]
IL-6	1.13 ± 0.13	1.68 ± 0.43	3.11 ± 1.17	136.45 ± 60.82	9.80 ± 3.48 [c]	5.99 ± 2.42 [c]
IL-10	2.24 ± 0.49	3.04 ± 1.14	1.46 ± 0.47	53.39 ± 22.25	5.09 ± 2.17 [c]	5.60 ± 2.86 [c]
IL-12p35	3.14 ± 0.82	1.69 ± 0.23	2.92 ± 1.07	8.35 ± 4.19	2.78 ± 1.42	2.59 ± 1.12
IL12/23p40	5.93 ± 2.86	4.79 ± 1.43	1.69 ± 0.81	74.18 ± 38.29	4.77 ± 1.28 [b]	6.21 ± 2.10 [c]

Treatment	Ctrl.	EPO	CIB	DSS + Ctrl.	DSS + EPO	DSS + CIB
Gene of interest						
IL-23p19	7.72 ± 1.93	6.36 ± 1.24	5.51 ± 1.47	3.91 ± 1.49	5.41 ± 1.90	6.54 ± 0.79
IL-18	3.36 ± 0.67	2.84 ± 0.73	2.13 ± 0.43	5.71 ± 0.64	2.50 ± 0.40 [a]	2.61 ± 0.31 [a]
IFN-γ	1.95 ± 0.52	4.20 ± 1.06	2.53 ± 1.50	27.54 ± 8.09	3.35 ± 0.99 [c]	3.20 ± 1.22 [c]
IL-4	0.81 ± 0.59	0.11 ± 0.11	0.25 ± 0.24	296.99 ± 142.26	5.67 ± 1.74 [c]	10.64 ± 7.30 [c]
IL-17A	2.74 ± 1.29	1.47 ± 0.37	1.38 ± 0.34	99.35 ± 22.39	18.81 ± 8.89 [b]	18.53 ± 9.70 [b]
TGF-ß	5.03 ± 1.43	6.87 ± 1.44	3.38 ± 0.89	22.82 ± 9.96	8.68 ± 1.17 [a]	6.90 ± 1.06 [b]
Foxp3	3.51 ± 0.85	5.32 ± 1.14	2.58 ± 0.80	110.15 ± 60.52	0.77 ± 0.16 [b]	1.15 ± 0.32 [c]
T-bet	0.84 ± 0.32	1.36 ± 0.36	0.78 ± 0.44	9.00 ± 5.06	0.19 ± 0.05 [c]	0.48 ± 0.18 [c]
GATA-3	0.22 ± 0.03	0.34 ± 0.09	0.15 ± 0.04	3.68 ± 1.44	0.11 ± 0.01 [c]	0.18 ± 0.07 [c]
RORγt	4.20 ± 1.75	4.92 ± 1.47	1.93 ± 0.69	1.63 ± 0.47	2.08 ± 0.78	0.91 ± 0.17

[a] $P < 0.05$ as compared to DSS + Ctrl.

[b] $P < 0.01$ as compared to DSS + Ctrl.

[c] $P < 0.001$ as compared to DSS + Ctrl.

Arbitrary units of target gene expression relative to the housekeeping gene Hprt.
